# Does high-frequency resistance exercise offer additional benefits to older adults? learnings from a randomized controlled trial

**DOI:** 10.1186/s13102-024-00975-6

**Published:** 2024-09-06

**Authors:** Qiaowei Li, Feng Huang, Yanling Cheng, Yalan Dai, Zhong Lin, Zhonghua Lin, Pengli Zhu

**Affiliations:** 1 Shengli Clinical Medical College of Fujian Medical University, Fuzhou, China; 2 Fuzhou University Affiliated Provincial Hospital, Fuzhou, China; 3 Fujian Provincial Institute of Clinical Geriatrics, Fuzhou, China; 4 Fujian Key Laboratory of Geriatrics, Fuzhou, China; 5 Fujian Provincial Center for Geriatrics, Fuzhou, China; 6 Department of Rehabilitation Medicine, Fujian Provincial Hospital, Fuzhou, China

**Keywords:** Dose-dependent, Resistance, Older, Randomized controlled trial

## Abstract

**Objective:**

Resistance exercise is an effective strategy to improve muscle strength in older adults. A limited-load resistance would be flexible and suitable for community-based training. It was unclear whether high-frequency resistance exercise offer additional benefits to older adults. Here, we aimed to examine the effect of limited-load resistance exercise among different frequency on muscle parameters in older adults.

**Methods:**

The current study was a single-blind, randomized controlled trial comparing the effectiveness of different-frequency resistance exercise in older adults. Change in skeletal muscle was estimated with a multi-frequency bioelectrical impedance analyzer. Demographics, physical examination, nutritional assessment, prealbumin and lymphocytes were also measured. Fisher’s precision probability test and baseline-adjusted generalized linear models were applied accordingly to analyze the influence of dose-different exercise on prevalence of sarcopenia, muscle parameters and body composition. A two-sided *p* value of < 0.05 was defined statistical significance.

**Results:**

The participants had a mean age of 71.96 years and close gender ratio. One hundred and twenty-seven participants (control 40; low-dose 46; high-dose 41) completed the 6-month exercise intervention. In contrast to control group, only high-dose exercise groups experienced improvements in muscle mass (0.66 kg, *p* < 0.001) and max grip strength (+ 2.17 kg, *p* < 0.001). There were significant dose-response effects of muscle mass (index), fat mass (index), max grip strength, 5-times sit to stand test, 6-minute walking test and visceral fat area (all *p*_trend_ <0.01).

**Conclusions:**

As the proved dose-dependent effect, current findings supported high-frequency limited-load resistance exercise applied and extended among older adults in community.

**Trial Registration:**

This study was registered at Chinese Clinical Trial Registry Network (ChiCTR2200062007, Registered on 19 July 2022).

**Supplementary Information:**

The online version contains supplementary material available at 10.1186/s13102-024-00975-6.

## What are the new findings

Dose-dependent effects of resistance training in improvement of global or segmental muscle mass in older adults.

Dose-dependent effects of resistance training in improvement of strength and physical function in older adults.

High-frequency limited-load resistance exercise has potential to be applied and extended among older adults in community.

**Supplementary Information:**

The online version contains supplementary material available at 10.1186/s13102-024-00975-6.

## Introduction

Normal ageing leads to gradual reduction in muscle mass and strength [1]. These declines in strength are related to significant deficits in functional ability [2]. Since decreased strength is correlated with subsequent functional decline and multimorbidity [3], improvements in strength would contribute to maintain independence and life quality the other way round. Resistance exercise has been recognized as an effective way to improve the neuromuscular function, max strength, power and the capacity to conduct multifunctional tasks in older adults, which may assist in preventing falls and frailty [4, 5].

The resistance exercise prescription is composed by series of variables including the intensity of training (load), frequency of training, the number of sets and repetitions in each training [6, 7]. Some of these variables have been verified in older adults, indicated that wide range of training regimens respond well to muscle strength and physical performance, even in those adopted relatively low intensities and frequencies [8, 9]. Although the American College of Sports Medicine (ACSM) recommends strength training twice a week both in young and older adults, some evidence implies once-weekly high-load resistance training would also be effective and even cost efficient [10]. However, investigation has identified issues with injury, illness and resistance training program the most commonly reasons why older people leave regular training [11]. Even though the improvement of muscle size and quality were mostly attributed to resistance training intensity [12], a high-load resistance may increase risk of injury and require more human resources to ensure a safe procedure. Yet a small-sample study has reported a high-load exercise has little progress on isometric strength and gait speed in postmenopausal women [13].

On the contrary, a limited-load resistance would be flexible and suitable for community-based training. However, to achieve an equivalent volume compared to high-load training, higher frequency may be required in limited-load resistance exercise. Previous finding suggested that advanced training frequency from once per week to 5 times per week achieved little gain in skeletal muscle mass and strength in young untrained men [14]. It was still unclear whether there exited a frequency-dependent response of limited-load exercise on improving or optimizing quantity and quality of muscle gains in older adults [13].

This study aimed to examine the effect of limited-load resistance exercise among different frequency on muscle parameters in older adults. Those results will assist to provide further specific evidence and rationales when prescribing flexible and efficient resistance training task for the older adults in community.

## Methods

### Study design

The current study was a randomized controlled trial comparing the effectiveness of different-frequency limited-load resistance exercise in independent older adults. The study was blinded only to outcome evaluators, data monitors and statistical analysts. The primary and secondary outcomes were assessed at baseline and after the 24-week resistance training. The study was approved by Fujian Provincial Hospital Ethics Committee and complied with the World Medical Association Declaration of Helsinki. Signed informed consents were obtained from all participants. CONSORT guidelines for reporting randomized trials were applied. The study was registered at Chinese Clinical Trial Registry Network as ChiCTR2200062007 on 19th July 2022.

### Participants and randomization

The inclusion criteria for this study were defined as follows: (1) Age range from 60 to 85 years; (2) Ability to complete the 400-m walk test within 15 min without sitting, the help with another person or the use of a walker; (3) Willingness to conduct study-related exercise plan. The exclusion criteria were: acute cardiac event, uncontrolled arrhythmias, acute heart failure, implantation of pacemakers or defibrillators, edema (would affect body composition measurement), asthma, cognitive dysfunction, neurologic disease or physical restrictions to perform exercise or assessment. Volunteer were recruited by posters in the hospital, WeChat advertisements, and physician referrals from July 2021. In total, 149 participants were enrolled, 70 (47.0%) of whom were male. Computerized randomization was performed to assign enrolled participants (1:1:1) into 3 arms: health education control group (performing resistance training not more than once a week), low-dose group (performing resistance training 2–3 times a week), and high-dose group (performing resistance training 4–5 times a week). Further baseline assessment and intervention will be performed within 2 weeks of assignment.

### Outcomes

The primary outcome was change in appendicular skeletal muscle index (ASMI) from baseline to 24 weeks. Second outcomes included other body composition parameters (such as, segmental skeletal mass of limbs and trunks, fat mass, visceral fat area and whole-body phase angle), indicators of muscle strength and physical function used in old adults.

### Intervention

All participants were required to take a part in a geriatric rehabilitation salon in the first two weeks, where geriatric physician and clinical physiotherapists systematically propagated exercise related knowledge for older population. The themes of this salon included the effect of resistance exercise for older adults, how to conduct upper limb resistance exercise, how to progressively conduct lower limb resistance exercise, how to train core muscle group, how to relax after resistance exercise and distinguish exercise-related pain. The control group subsequently was distributed with training brochures, and asked to practice no more than once a week at home. The progress was controlled by themselves according to the knowledge learning from the geriatric rehabilitation salon. The records of home-based exercise were submitted to reviewed through Wechat app every week to reduce memory bias. During the 24 weeks, the low-dose group was arranged with twice a week center-based training and recommended home-based self-practice not more than once a week. The high-dose group was arranged with four times center-based training and recommended home-based self-practice not more than once per week. All center-based training sessions were performed under supervision or observation by physical professionals to ensure compliance with the training programs and safety of the older adults. After a warm up of 5–10 min on the treadmill, participants performed resistance exercise using a combination of machines and free weights. Both training groups performed the same program performed with standard illustration: biceps curl, dumbbell lateral raise, dumbbell shoulder press, bent-over dumbbell row, push-up or its variations (wall push-up, raised push-up, raised push-up), (dumbbell) squat, standing side/straight leg raise, and glute bridge. Each set was performed for 12–20 repetitions, with a target rate of perceived exertion ranging from 11 to 13. The trainer will assess and record participants’ rate of perceived exertion at each session. The load for each exercise was up-graded every 4 weeks. According to previous load, the augmentation for upper and lower limbs were 2–5% and 5–10%, respectively [15]. At the end of each session, flexibility exercises targeted all major muscle groups were performed.

### Outcome measurements

The participants should not eat for 4 h before the tests and refrain from alcohol for 12 h. In addition, the participants should also be advised not to work out at the gym 8 h prior to the procedure.

### Demographics and nutritional assessments

Living conditions, smoking history, alcohol consumption, medical history and medications were assessed by questionnaires. Anthropometric data including height, weight, blood pressure, waist and hip circumference were measured. Nutritional status was assessed by the Short-form Mini Nutritional Assessment (MNA-SF) [16]. Blood samples were collected in the morning after an overnight fast [17, 18], then prealbumin and lymphocytes were measured using the immune-nephelometry (Siemens, Erlangen, Germany) and the automated cell counter (Beckman Coulter, Fullerton, USA), respectively.

### Muscle strength and physical function

The short physical performance battery (SPPB) was administered to assess physical performance of older adults. The total score (ranging from 0 to 12 points) was calculated by three tests consisted of usual-pace gait speed, a 5-times sit-to-stand test, and a balance test [19]. Handgrip strength [20] was assessed using a dynamometer (Jamar Plus, United States) in a sitting position. The maximum value of three measurements in the dominant hand was recorded for analysis. The 6-minute walking test (6MWT) performed according to the technical standard proposed by European Respiratory Society/American Thoracic Society [21].

### Body composition measurement

Change in body composition was estimated with a multi-frequency bioelectrical impedance analyzer, InBody770 (Biospace, Korea). Prior to the procedure, all jewelry should be removed, and the skin should be dry. During the assessment, participants stood barefoot on the platform of the device with the soles of their feet on the electrodes. They then grasped the handles of the unit with their thumb and fingers to maintain direct contact with the electrodes and remained still for about 1 min while keeping their elbows fully extended and their shoulder joint abducted to an angle of about 30°. The measurements included total skeletal mass, segmental skeletal mass of limbs and trunks, fat mass, visceral fat area and whole-body phase angle. The instrument has been validated in Chinese older adults [22]. ASMI and fat mass index (FMI) were calculated as appendicular skeletal mass divided by height squared and fat mass divided by height squared, respectively.

### Definition of sarcopenia

Thresholds for low ASMI, low handgrip strength and slow gait speed were identified according to the 2019 AWGS consensus [23] and Chinese sarcopenia expert consensus [24]. Sarcopenia was defined as low ASMI plus low handgrip strength and/or slow gait speed. Pre-sarcopenia was defined as low ASMI without a decline in handgrip strength or gait speed.

### Statistical analysis

PASS software was used to calculate the sample size. The estimated means (standard deviation) of ASMI was 6.14 (1.13) kg/m^2^ as reported by an Asian cross-sectional study [25]. And we anticipated to detect a 10 and 20% difference in ASMI among the three groups, respectively. Thus a priori sample size calculations at a power of 80% and a significant level of 0.9, required recruiting at least 28 older adults for each group accounting a drop-out rate of 20%.

Demographic and geriatric characteristics of all participants were summarized as mean ± standard deviation (SD) or frequency and percentage. Independent 2-sample Student *t* tests or chi-square analysis was conducted to assess baseline differences between older adults completed the follow-up and who withdrew from the study.

We used analysis of variance (ANOVA), chi-square analysis or Fisher’s precision probability test to compare the baseline demographic, anthropometric, blood pressure and nutritional status of different groups.

Fisher’s precision probability test and baseline-adjusted generalized linear models were applied accordingly to analyze the influence of dose-different exercise on prevalence of sarcopenia, muscle parameters and body composition. When significant trends were observed, a Dunnett-Hsu post hoc assessment was conducted. The influence of dose-different exercise on muscle parameters and body composition were further analyzed in sex stratification. Student *t* tests for paired data was used to assess changes within the group from baseline to 24-week follow-up. Statistical analyses were performed using R, version 4.0.4 (http://www.r-project.org). A two-sided *p* value of < 0.05 was defined statistical significance.

## Results

**Fig. 1:**
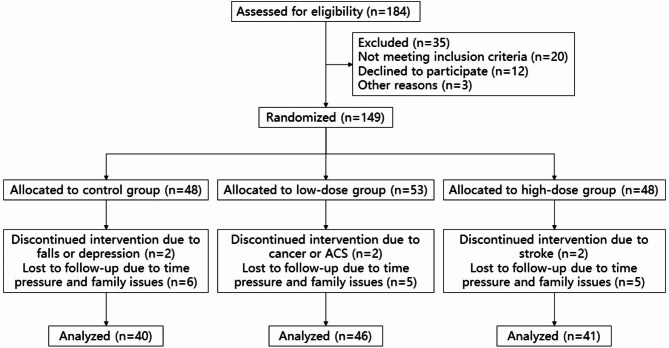
Flow chart of study. ACS, acute coronary syndrome

A total of 184 potential participants were invited to the center for detailed explanations of the study, questionnaires, and 400-meter walking test. Eligibility was confirmed after screening for inclusion and exclusion criteria. Consequently, 149 older adults were accepted to the study (flow chart of study was shown in Fig. 1).

A sensitivity analysis was conducted to compare baseline measurements between the 22 older adults who withdrew from the study prior to follow-up and the 127 older adults completed the study (Supplementary Table 1). Reasons for withdrawal from the study were mostly time pressures or family issues (Fig. 1). The were no significant differences between study participants and older adults who withdrew from the study in age, gender, habits, living conditions, blood pressure, nutritional status (MNA-SF, lymphocyte count and prealbumin), chronic disease, falls history or medication related to muscle synthesis. Older adults attended a mean (SD) of 98.10% (13.38%) of the 24-week exercise sessions (median adherence rate 100%; minimum, 54.17%) in low-dose group, and a mean (SD) of 93.57% (7.81%) of the 24-week exercise sessions (median adherence rate 96.88%; minimum, 76.04%) in high-dose group.

The demographic, anthropometric, blood pressure and nutritional status data were listed in Table 1. Overall, the participants had a mean age of 71.96 years and close gender ratio. Absolute changes in muscle parameters and body composition from baseline to follow-up were assessed within groups (Supplementary Table 2). Control group participants experienced significant decrease in total skeletal muscle mass (-0.36 kg) and ASMI (-0.19 kg/m^2^), an increase in phase angel (0.1°). Both of the low-dose and high-dose exercise groups experienced significant decrease in fat mass (-1.26 kg and − 1.65 kg), FMI (-0.5 kg/m^2^ and − 0.63 kg/m^2^) and visceral fat area (-7.54 cm^2^ and − 10.01 cm^2^).

**Table 1:** Demographic, anthropometric, blood pressure and nutritional status of baseline

Variable	All (*N* = 127)	Control (*N* = 40)	Low-dose (*N* = 46)	High-dose (*N* = 41)	*p*
Age (yr)	71.96 ± 4.54	71.30 ± 4.18	72.87 ± 4.50	71.59 ± 4.87	0.228
Height (cm)	162.90 ± 8.50	162.01 ± 9.60	163.34 ± 8.06	163.28 ± 7.98	0.730
Weight (kg)	62.92 ± 10.11	63.88 ± 11.61	62.70 ± 8.56	62.22 ± 10.33	0.752
Waist hip ratio	0.92 ± 0.07	0.93 ± 0.07	0.90 ± 0.07	0.92 ± 0.07	0.265
Systolic blood pressure (mmHg)	132.57 ± 19.55	133.83 ± 21.43	134.85 ± 19.50	128.80 ± 17.49	0.317
Diastolic blood pressure (mmHg)	75.98 ± 11.43	75.48 ± 11.44	76.72 ± 10.92	75.63 ± 12.20	0.860
MNA-SF sore	12.94 ± 1.38	13.03 ± 1.39	12.96 ± 1.35	12.83 ± 1.45	0.813
Lymphocyte count (10^9^/L)	2.18 ± 0.87	2.34 ± 0.84	2.24 ± 1.08	1.96 ± 0.58	0.130
Prealbumin (g/L)	266.02 ± 37.67	276.87 ± 39.69	263.21 ± 32.79	259.19 ± 39.53	0.090
Gender (male)	62(48.80)	19(47.50)	24(52.20)	19(46.30)	0.845
Living alone	23(18.1)	8(20.0)	7(15.2)	8(19.5)	0.815
House with stairs	61(48.0)	17(42.5)	25(54.3)	19(46.3)	0.529
Smoking	20(15.7)	9(22.5)	6(13.0)	5(12.2)	0.365
Alcohol consumption	8(6.3)	3(7.5)	2(4.3)	3(7.3)	0.864
Hypertension	72(56.7)	25(62.5)	32(69.6)	15(36.6)	**0.006**
Diabetes mellitus	40(31.5)	18(45.0)	15(32.6)	7(17.1)	**0.025**
Falls history	14(11.0)	5(12.5)	5(10.9)	4(9.8)	0.939
Medication					
Statin	46(36.2)	12(30.0)	19(41.3)	15(38.1)	0.552
Metformin	17(13.4)	9(22.5)	6(13.0)	2(4.9)	0.067
Insulin	7(5.5)	2(5.0)	4(8.7)	1(2.4)	0.500

Data in parentheses are percentage. *p* stands for ANOVA, chi-square analysis or Fisher’s precision probability test of different groups. MNA-SF, Mini nutritional assessment short-form

Compared to the control group, the low-dose group had a significantly improvement in change of ASMI (adjusted MD 0.13 kg/m^2^, 95%CI: 0.04 to 0.22, *p* = 0.006), and the high-dose had a further improvement over low-dose group (adjusted MD 0.22 kg/m^2^, 95%CI: 0.13 to 0.32, *p* < 0.001; adjusted MD compared to low-dose 0.10 kg/m^2^, 95%CI: 0.01 to 0.19, *p* = 0.036). The high-dose also added benefits in 5-times sit to stand test (Supplementary Table 3). Stratified Analysis showed only female participants gained ASMI improvements in low-dose groups (adjusted MD 0.17 kg/m^2^, 95%CI: 0.03 to 0.30, *p* = 0.015). Both female and male participants achieved ASMI improvements in high-dose groups. There seemed no sex difference among fat mass, FMI and visceral fat area in the effect of resistance training (Supplementary Tables 4–5).

**Fig. 2:**
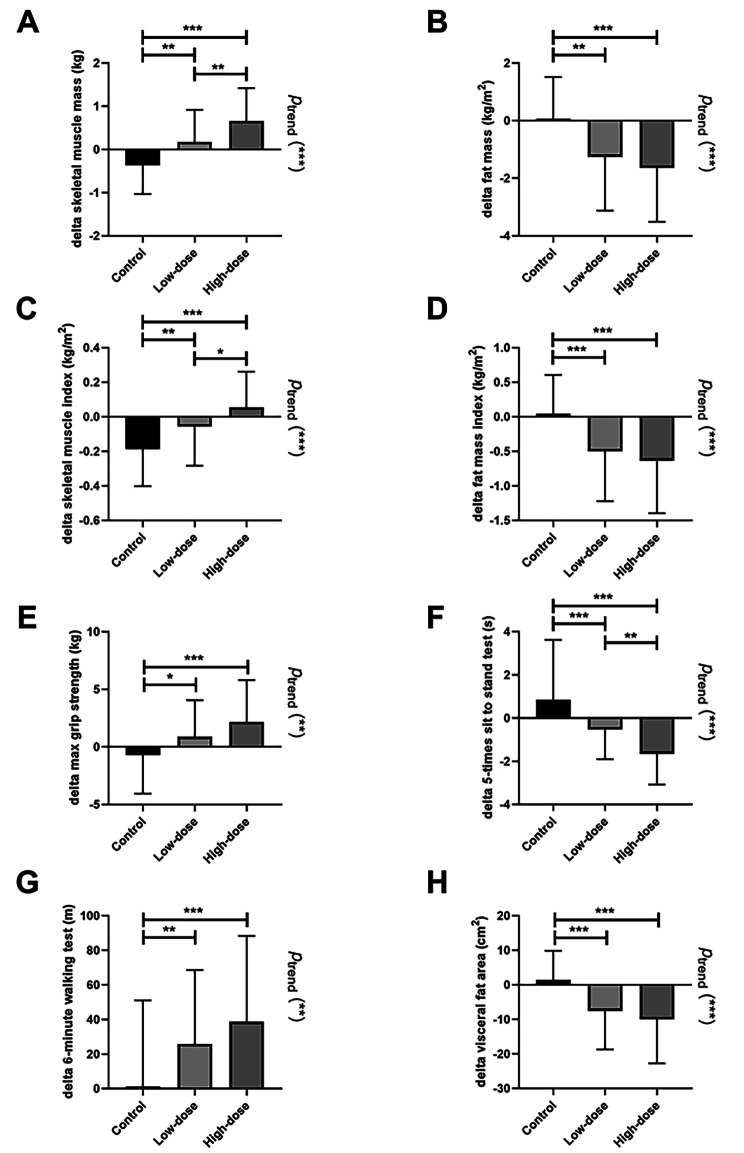
Absolute changes in muscle mass, muscle function, body fat and visceral fat after dose-different exercise. ASMI, appendicular skeletal muscle mass index. *p*_trend_, linear trend analysis assessed whether the change was exercise dose-dependent *p < 0.05; **p < 0.01; ***p < 0.001

We further tested whether those changes were linearly correlated with the dose of exercise intervention (Fig. 2). There were significant dose response effects of muscle mass (index), fat mass (index), max grip strength, 5-times sit to stand test, 6MWT and visceral fat area. The post hoc analyses showed each succeeding level of exercise energy expenditure added another significant improvement in total skeletal muscle mass, ASMI and 5-times sit to stand test.

**Fig. 3:**
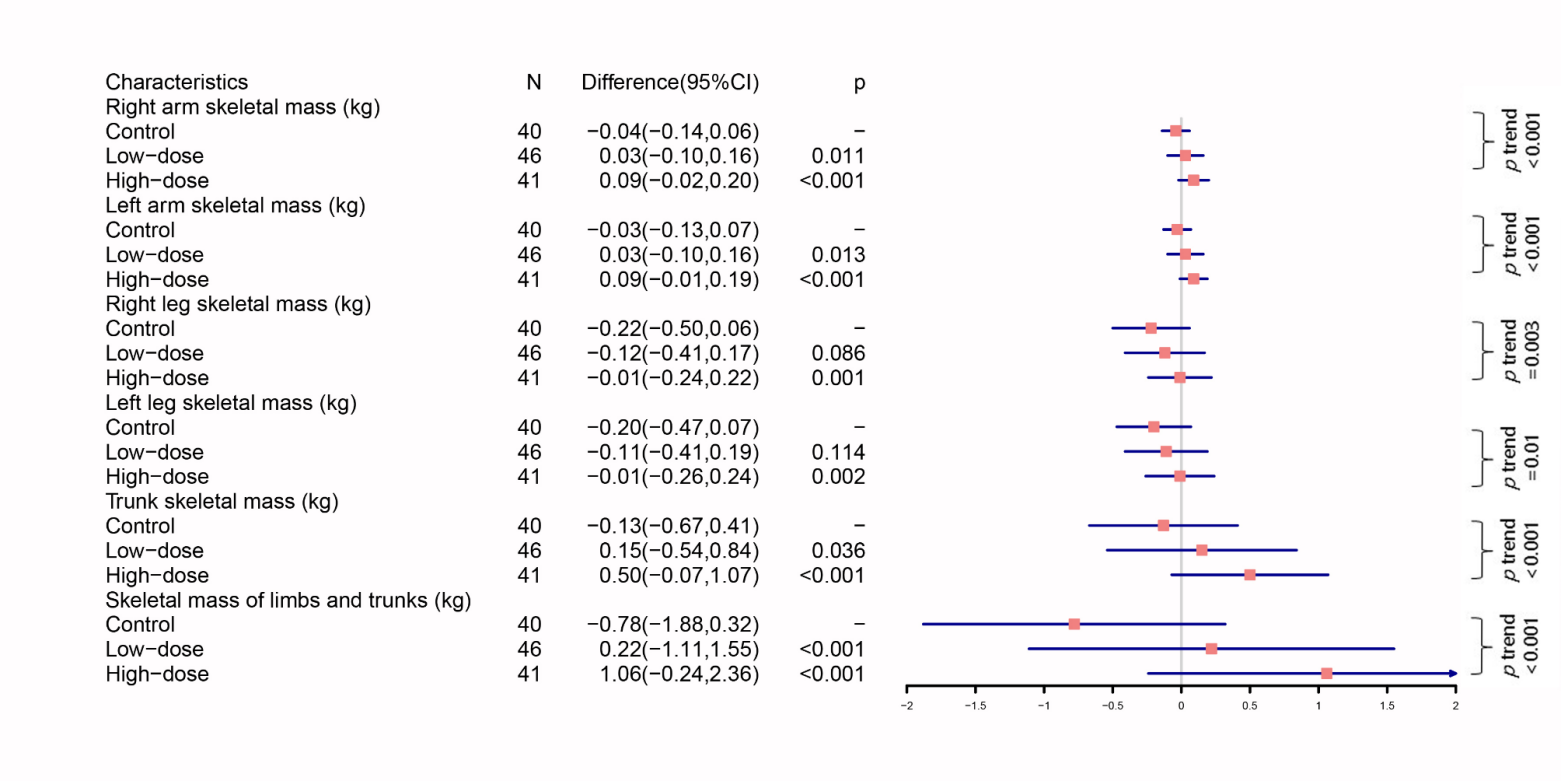
Segmental analysis of changes in skeletal muscle mass. *p*, compared to control group. *p*_trend_, linear trend analysis assessed whether the change was exercise dose-dependent

The segmental analyses showed that compared to control group, only high-dose exercise leaded to a significant change in skeletal muscle mass of lower limbs (Fig. 3). All segmental skeletal muscle mass appeared dose-dependent changes.

**Fig. 4:**
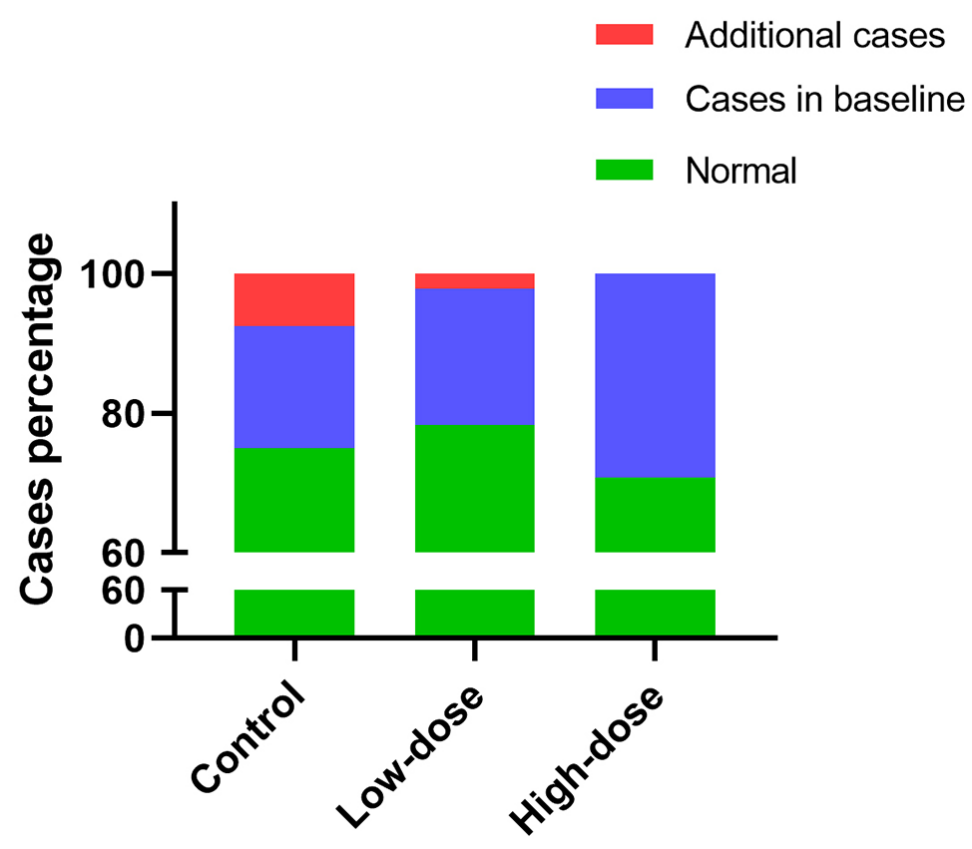
Change of participants with sarcopenia or pre-sarcopenia. Cases, sarcopenia or pre-sarcopenia

We also summarized the prevalence of sarcopenia or pre-sarcopenia in participants. In control group, the number of cases increased from 7 to 10. In the low-dose exercise group, the number of cases increased by 1 (from 9 to 10). However, the number maintained at 12 from allocation to the end of follow-up in the high-dose exercise group. The changes in prevalence of sarcopenia or pre-sarcopenia among the three groups were not significantly different (Fig. 4).

## Discussion

Current study examined the effect of different frequency, limited intensity, total-body resistance exercise on muscle parameters and body composition in older adults. Significant exercise frequency-dependent increase was observed in change of muscle mass, strength and physical function. In segmental analysis of skeletal muscle mass, only high-dose exercise resulted in significant improvement of lower limbs muscle mass equivalent. Moreover, control group experienced light increases in body fat mass and visceral fat area whereas the intervention groups experienced significant decrease in those two indicators.

Numerous investigations have identified resistance exercise an effective strategy for older adults, which may elicit significant improvements in muscular strength capacity [26, 27]. There seemed to be a consensus high-load resistance training leaded to greater strength gains and hypertrophy than can moderate-intensity resistance training in older adults [28–30]. However, the effectiveness of training volume (e.g., frequency, period, number of sets, number of repetitions) for strength improvement is inconsistent across investigations. Previous meta-regression included twenty-four studies illustrated that the training volume of resistance exercise had little impact on muscle morphology [31].

As ACSM [15] recommends the minimal frequency of strength training to be twice a week, most studies focused on a minimal frequency. Taaffe et al [32] compared the effect of higher training frequency with recommence. However, they concluded that a weekly or biweekly high-load resistance training is equally effective to three sessions per week. Furthermore, DiFrancisco-Donoghue et al [33] reported resistance exercise once weekly in wellness older adults achieved equal muscle strength improvement with those in twice-a-week program. Of note, those findings should be interpreted with caution as the range of training frequencies mentioned in the study was still narrow (not more than three sessions per week). On the other hand, with high prevalence of multimorbidity [34, 35], supervision during exercise was of necessity for the majority older adults, not to mention high-load training which would consume much more resources when applied in older adults. Therefore, current study comparing well-tolerated resistance exercise in different frequency rather than intensity may improve the validity of the results and increase the feasibility of practice.

The results of this study compared favorably with those of Farinatti et al [36], who compared the effect of three different training frequencies on strength and functional performance in physically active women aged 60 years and older. They concluded that compared to standard training volume, both strength and functional ability improved to a greater extent with a higher-frequency training protocol. Whereas the study only analyzed 41 healthy women, similar results were achieved in current experiment with 127 mix-gender older adults aged 60–85. As the contribution of resistance training volume to physiological adaptations have been well demonstrated, it was easy to hypothesize that higher frequency would achieve more strength gains compared to twice weekly commonly proposed. In present lower intensity program, high-dose resistance training with a frequency above 3 times per week elicited greater total skeletal muscle mass gains and 5TST improvement when compared to low-dose.

Our results also presented a sex difference response of resistance training in muscle mass. Glycolytic fibers were more abundant in men than in women [37], which could account for the differential sensitivity to the resistance training between sexes. We speculated the limited-intensity resistance exercise affected oxidative fibers to a great extent than glycolytic ones. Thus, women could benefit from low-dose training, while men could only gain muscle mass through high-dose resistance training. In this case, older males were particularly recommended to adopt high-dose resistance training for a great improvement in muscle.

A recent review on the resistance exercise for sarcopenia proposed a programme performed with a relatively high degree of effort for 1–3 sets of 6–12 repetitions [38]. However, it may be difficult to implement and costly to supervise weekly high intensity exercise in older patients. Therefore, the resistance exercise for the treatment of sarcopenia within the research world has yet to translate into consistent provision in clinical practice. Based on the incidence of sarcopenia in our research, we suspected there may be a trend that high-dose exercise reduced the incidence of sarcopenia or pre-sarcopenia cases. Those results indicated additional benefits of high-dose limited-load resistance training in population closer to the real world. A major subset of adults over the age of 65 is now classified as having sarcopenic obesity, a high-risk geriatric syndrome predominantly observed in an ageing population that is at risk of synergistic complications from both sarcopenia and obesity [39]. Accordingly, priority outcomes for older adult’s exercise program are not only to increase skeletal mass but also decrease fat mass. Our programme showed a satisfied effect on fat mass and visceral fat area in both low-dose and high-dose, which further supported it to be applied in older adults.

The primary limitation of our study is that we did not measure dynamic or isokinetic muscle strength of major muscle groups by instruments as results of the training. The proxy of muscle strength currently used, grip strength and 5TST, were recommended by Asian and European Working Group of Sarcopenia and well-documented of correlation with lower extremity muscle power or knee extension torque [23, 40]. Moreover, the efficiency of our experiment was improved because such indicators were adopted. Secondly, dietary intake, especially protein-rich food consumption or any protein/branched chain amino acid supplements not assessed may induce bias to the results. Lastly, the study was single-centered, and broadly including community-dwelling population with comorbidity and polypharmacy, which may limit the application of its findings to older adults in other situations. Future larger and multiple center studies were need to confirm current findings.

In conclusion, the study demonstrated dose-dependent effects of resistance training in improvement of global or segmental muscle mass, strength and physical function. The results supported high-frequency limited-load resistance exercise applied and extended among older adults in community.

## Electronic supplementary material

Below is the link to the electronic supplementary material.

Supplementary Material 1

Supplementary Material 2

Supplementary Material 3

## Data Availability

Data is available within permission from corresponding author.

## Abbreviations

ACSM: American College of Sports Medicine
ASMI: Appendicular skeletal muscle index
MNA-SF: Short-form Mini Nutritional Assessment
SPPB: the short physical performance battery
6MWT: the 6-minute walking test
FMI: Fat mass index
