# Supplementary material for: Does high-frequency resistance exercise offer additional benefits to older adults? learnings from a randomized controlled trial
Source: BMC Sports Sci Med Rehabil. 2024 Sep 6;16:186. doi: 10.1186/s13102-024-00975-6 (PMC11378542; doi:10.1186/s13102-024-00975-6)
Supplement: Supplementary file 2 — Supplementary Material 2 [file 13102_2024_975_MOESM2_ESM.docx]

**Supplement 1. Original Protocol**

Introduction

Normal ageing leads to gradual reduction in muscle mass and strength[1, 2]. These declines in strength are related to significant deficits in functional ability[2]. Since decreased strength is correlated with subsequent functional decline and multimorbidity[3], improvements in strength would contribute to maintain independence and life quality the other way round. Resistance exercise has been recognized as an effective way to improve the neuromuscular function, max strength, power and the capacity to conduct multifunctional tasks in older adults, which may assist in preventing falls and frailty[4, 5].

The resistance exercise prescription is composed by series of variables including the intensity of training (load), frequency of training, the number of sets and repetitions in each training[6, 7]. Some of these variables have been verified in older adults, indicated that wide range of training regimens respond well to muscle strength and physical performance, even in those adopted relatively low intensities and frequencies[8, 9]. Although the American College of Sports Medicine (ACSM) recommends strength training twice a week both in young and older adults, some evidence implies once-weekly high-load resistance training would also be effective and even cost efficient[4, 6]. However, investigation has identified issues with injury, illness and resistance training program the most commonly reasons why older people leave regular training[10]. Even though the improvement of muscle size and quality were mostly attributed to resistance training intensity[11], a high-load resistance may increase risk of injury and require more human resources to ensure a safe procedure. Yet a small-sample study has reported a high-load exercise has little progress on isometric strength and gait speed in postmenopausal women[12].

On the contrary, a limited-load resistance would be flexible and suitable for community-based training. However, to achieve an equivalent volume compared to high-load training, higher frequency may be required in limited-load resistance exercise. Previous finding suggested that advanced training frequency from once per week to 5 times per week achieved little gain in skeletal muscle mass and strength in young untrained men [13]. It was still unclear whether there exited a frequency-dependent response of limited-load exercise on improving or optimizing quantity and quality of muscle gains in older adults.

The primary outcome is change in appendicular skeletal muscle index (ASMI) from baseline to 24 weeks.

The second outcomes include other body composition parameters (such as, segmental skeletal mass of limbs and trunks, fat mass, visceral fat area and whole-body phase angle), indicators of muscle strength and physical function used in old adults.

Methodology

Registration

The study protocol has been granted approval by Fujian Provincial Hospital Ethics Committee. The study adheres to the Helsinki Declaration and is prospectively registered with Chinese Clinical Trial Registry Network (https://www.chictr.org.cn/) identifier: # ChiCTR2200062007.

Study design and setting

This study is a single-center, randomized controlled trial comparing the effectiveness of different-frequency limited-load resistance exercise in independent older adults. The study will be blinded only to outcome evaluators, data monitoring and statistical analysts. The primary and secondary outcomes are assessed at baseline and after the 24-week resistance training.

Participants and eligibility criteria

The inclusion criteria for this study are defined as follows: 1) Age range from 60-85 years; 2) Ability to complete the 400-m walk test within 15 min without sitting, the help with another person or the use of a walker; 3) Willingness to conduct study-related exercise plan. The exclusion criteria are: acute cardiac event, uncontrolled arrhythmias, acute heart failure, implantation of pacemakers or defibrillators, edema (would affect body composition measurement), asthma, cognitive dysfunction, neurologic disease or physical restrictions to perform exercise or assessment. Volunteer will be recruited by posters in the hospital, WeChat advertisements, and physician referrals from July 2021.

Recruitment, randomization and allocation

The study aims to recruit at least 84 older adults. Recruitment strategies include posters in the hospital, WeChat advertisements, and physician referrals. Potential participants will be invited to the center for detailed explanations of the study. Eligibility is confirmed after screening for inclusion and exclusion criteria.

The participants will be 1:1 randomly assigned into into 3 arms: health education control group (performing resistance training not more than once a week), low-dose group (performing resistance training 2-3 times a week), and high-dose group (performing resistance training 4-5 times a week). A computerized random number generator will be used to create the randomization sequence. The randomization result will be transferred to allocation cards and sealed in opaque envelopes. A designated staff out of the research will be responsible to generate and save randomization sequence. Further baseline assessment and intervention will be performed within 2 weeks of assignment. The study will be blinded only to outcome evaluators, data monitoring and statistical analysts.

Data collection, monitoring, and management

Participants will be assessed at 2 testing periods: baseline (week 0) and 24-week follow-up. Data will be collected on a case report form, comprehensive geriatric assessment and body composition detection at each assessment.

Data required for analysis will then be entered into and stored within Geriatric Rehabilitation Management System on Fujian Provincial Institute of Clinical Geriatrics by one operator. Paper data will be kept in lockable filing cabinets throughout the study period and 3 years thereafter. Electronic files will be password protected and only available to members of the investigation team approved by Fujian Provincial Hospital Ethics Committee.

Intervention

All participants will be required to take a part in a geriatric rehabilitation salon in the first two weeks, where geriatric physician and clinical physiotherapists systematically propagated exercise related knowledge for older population. The themes of this salon include the effect of resistance exercise for older adults, how to conduct upper limb resistance exercise, how to progressively conduct lower limb resistance exercise, how to train core muscle group, how to relax after resistance exercise and distinguish exercise-related pain.

All center-based training sessions will be performed under supervision or observation by physical professionals to ensure compliance with the training programs and safety of the older adults. After a warm up of 5 -10 min on the treadmill, participants perform resistance exercise using a combination of machines and free weights. Both training groups perform the same program performed with standard illustration: biceps curl, dumbbell lateral raise, dumbbell shoulder press, bent-over dumbbell row, push-up or its variations (wall push-up, raised push-up, raised push-up), (dumbbell) squat, standing side/straight leg raise, and glute bridge. Each set was performed for 12-20 repetitions, with a target rate of perceived exertion ranging from 11 to 13. The trainer will assess and record participants’ rate of perceived exertion at each session. The load for each exercise will be up-graded every 4 weeks. According to previous load, the augmentation for upper and lower limbs were 2–5 % and 5–10 %, respectively[14]. At the end of each session, flexibility exercises targeted all major muscle groups will be performed.

During the 24 weeks, the control group will be subsequently distributed with training brochures, and asked to practice no more than once a week at home. The progress is controlled by themselves according to the knowledge learning from the geriatric rehabilitation salon. The records of home-based exercise will be submitted to reviewed through Wechat app every week to reduce memory bias.

The low-dose group will be arranged with twice a week center-based training and recommended home-based self-practice not more than once a week.

The high-dose group will be arranged with four times center-based training and recommended home-based self-practice not more than once per week.

Compliance and safety

Compliance to the intervention will be calculated as the percentage of expected sessions attended over the study. All subjects are required to keep an exercise diary log to note the day, types and perceived exertion of each exercise session, as well as any adverse events during or after the exercise. The diary log will be uploaded through Geriatric Rehabilitation Management System on Fujian Provincial Institute of Clinical Geriatrics. The rehabilitator encouraged the continued completion of compliant training and the timely adjustment of the training intensity. Subjects were considered to have completed the intervention if they completed 75% of the training requirements. Adverse event will be recorded and assessed by medical staff. The subject will be referred to and handled by specialist if the event needs medical intervention, and the situation will be followed up.

Outcome measures

The participants should not eat for 4 hours before the tests and refrain from alcohol for 12 hours. In addition, the participants should also be advised not to work out at the gym 8 hours prior to the procedure.

**Body composition measurement**

Change in body composition is estimated with a multi-frequency bioelectrical impedance analyzer, InBody770 (Biospace, Korea). Prior to the procedure, all jewelry should be removed, and the skin should be dry. During the assessment, participants stood barefoot on the platform of the device with the soles of their feet on the electrodes. They then grasped the handles of the unit with their thumb and fingers to maintain direct contact with the electrodes and remained still for about 1 min while keeping their elbows fully extended and their shoulder joint abducted to an angle of about 30°. The measurements include total skeletal mass, segmental skeletal mass of limbs and trunks, fat mass, visceral fat area and whole-body phase angle. The instrument has been validated in Chinese older adults [15]. ASMI and fat mass index (FMI) will be calculated as appendicular skeletal mass divided by height squared and fat mass divided by height squared, respectively. Thresholds for low ASMI, low handgrip strength and slow gait speed are identified according to the 2019 AWGS consensus[16]. Sarcopenia is defined as low ASMI plus low handgrip strength and/or slow gait speed. Pre-sarcopenia is defined as low ASMI without a decline in handgrip strength or gait speed.

**Demographics and nutritional assessments**

Nutritional status

We use scores of Short form Mini Nutritional Assessment (MNA-SF)[17] to classify subjects as normal nutritional status, with a risk of malnutrition or malnutrition. The MNA-SF is a questionnaire containing 6 questions, collecting subjects' appetite, appetite, activity ability, psychological trauma, cognitive function, body mass index/calf circumference.

Cognitive function

The Mini-mental State Examination cognitive scale[18] will be applied to conduct a comprehensive examination of cognitive function through a 30-item questionnaire, including time and place orientation ability, memory, attention and calculation ability, language use and comprehension ability, and recall ability. Cognitive dysfunction was defined as a Mini-Mental State Examination score less than 27.

6-minute walking test (6MWT)

6MWT is commonly used to assess the functional status of patients with severe cardiopulmonary disease. The testing technique is based on standards proposed by the European Respiratory Association/American Thoracic Association[19]. The test is conducted in a corridor with an indoor length exceeding 30 m, with two eye-catching traffic cones at the starting and ending points at a distance of 20 m. The subjects will rest for 15 minutes before informed to walk as quickly and comfortably as possible to shuttling between the two cones. The absolute distance, heart rate, blood pressure, blood oxygen saturation, and perceived fatigue level will be recorded.

Gait speed

Gait speed is tested in a 4-meter trail reserved 1 meter in front of the starting point. The examiner will inform the subjects to walk through the entire trail at their daily speed, record the time taken, and convert it into speed.

5 times sit to stand test (5TST)

The 5TST is used to evaluate lower limb strength[20], which requires subjects sit on an armless chair with a height of 45.7cm firstly with hands crossing in front of their chest. After hearing the "start" command, the subjects quickly complete 5 times of standing up and sitting down. The examiner will record the time from the beginning to the last time they sit down and contact with the chair surface.

Max grip strength

The subjects are seated, elbow flexed in 90 degrees. The strength of both upper limbs will be measured using a hydraulic grip strength meter (Jamar Plus, USA), for three times, with a grip interval of more than 15 seconds. And the maximum value was recorded. The reading is accurate one decimal place.

Sample size calculation

PASS software was used to calculate the sample size. The estimated means (standard deviation) of ASMI was 6.14 (1.13) kg/m^2^ as reported by an Asian cross-sectional study[21]. And we anticipated to detect a 10 and 20% difference in ASMI among the three groups, respectively. Thus a priori sample size calculations at a power of 80% and a significant level of 0.9, required recruiting at least 28 older adults for each group accounting a drop-out rate of 20%.

Statistical analysis

Demographic and geriatric characteristics of all participants will be summarized as mean ± standard deviation (SD) or frequency and percentage. Independent 2-sample Student *t* tests or chi-square analysis is conducted to assess baseline differences between older adults completed the follow-up and who withdrew from the study.

We will use analysis of variance (ANOVA), chi-square analysis or Fisher's precision probability test to compare the baseline demographic, anthropometric, blood pressure and nutritional status of different groups.

Fisher’s precision probability test and baseline-adjusted generalized linear models are applied accordingly to analyze the influence of dose-different exercise on prevalence of sarcopenia, muscle parameters and body composition. When significant trends will be observed, a Dunnett-Hsu post hoc assessment will be conducted. Student *t* tests for paired data will be used to assess changes within the group from baseline to 24-week follow-up. Statistical analyses are performed using R, version 4.0.4 (http://www.r-project.org). A two-sided *p* value of <0.05 is defined statistical significance.

Discussion

Older adults are often limited by their personal health, family things and social support to maintain mid-high-intensity training over time. Our study tries to compare the limited-intensity resistance exercise among different frequency on body composition. The results may support the utility of high-frequency lower-intensity resistance exercise among older adults in community.

Conclusion

We proposed a three-arm RCT plan to help identify a high-frequency resistance exercise offer additional benefits to older adults. Elucidating the effect of this plan would confirm the limited-intensity high-frequency resistance exercise could be extended and emphasize in a huger range of old population.

References

1. Larsson L, Grimby G, Karlsson J. Muscle strength and speed of movement in relation to age and muscle morphology. J Appl Physiol Respir Environ Exerc Physiol. 1979;46:451-6.

2. Murray MP, Gardner GM, Mollinger LA, Sepic SB. Strength of isometric and isokinetic contractions: knee muscles of men aged 20 to 86. Phys Ther. 1980;60:412-9.

3. Syddall H, Cooper C, Martin F, Briggs R, Aihie Sayer A. Is grip strength a useful single marker of frailty? Age Ageing. 2003;32:650-6.

4. Taaffe DR, Duret C, Wheeler S, Marcus R. Once-weekly resistance exercise improves muscle strength and neuromuscular performance in older adults. J Am Geriatr Soc. 1999;47:1208-14.

5. Fiatarone MA, Marks EC, Ryan ND, Meredith CN, Lipsitz LA, Evans WJ. High-intensity strength training in nonagenarians. Effects on skeletal muscle. JAMA. 1990;263:3029-34.

6. Feigenbaum MS, Pollock ML. Prescription of resistance training for health and disease. Med Sci Sports Exerc. 1999;31:38-45.

7. Galvao DA, Taaffe DR. Resistance exercise dosage in older adults: single- versus multiset effects on physical performance and body composition. J Am Geriatr Soc. 2005;53:2090-7.

8. Taaffe DR, Pruitt L, Pyka G, Guido D, Marcus R. Comparative effects of high- and low-intensity resistance training on thigh muscle strength, fiber area, and tissue composition in elderly women. Clin Physiol. 1996;16:381-92.

9. Vincent KR, Braith RW. Resistance exercise and bone turnover in elderly men and women. Med Sci Sports Exerc. 2002;34:17-23.

10. Burton E, Hill AM, Pettigrew S, Lewin G, Bainbridge L, Farrier K, et al. Why do seniors leave resistance training programs? Clin Interv Aging. 2017;12:585-92.

11. Lopez P, Pinto RS, Radaelli R, Rech A, Grazioli R, Izquierdo M, et al. Benefits of resistance training in physically frail elderly: a systematic review. Aging Clin Exp Res. 2018;30:889-99.

12. CARNEIRO MAS, KASSIANO W, OLIVEIRA-Jц NIOR G, SOUSA JFR, CYRINO ES, ORSATTI FBL. Effect of Different Load Intensity Transition Schemes on Muscular Strength and Physical Performance in Postmenopausal Women. Medicine & Science in Sports & Exercise. 2023;55:1507-23.

13. Franco CMC, Carneiro MAS, de Sousa JFR, Gomes GK, Orsatti FL. Influence of High- and Low-Frequency Resistance Training on Lean Body Mass and Muscle Strength Gains in Untrained Men.

14. American College of Sports M, Chodzko-Zajko WJ, Proctor DN, Fiatarone Singh MA, Minson CT, Nigg CR, et al. American College of Sports Medicine position stand. Exercise and physical activity for older adults. Med Sci Sports Exerc. 2009;41:1510-30.

15. Wang H, Hai S, Cao L, Zhou J, Liu P, Dong BR. Estimation of prevalence of sarcopenia by using a new bioelectrical impedance analysis in Chinese community-dwelling elderly people. BMC Geriatr. 2016;16:216.

16. Chen LK, Woo J, Assantachai P, Auyeung TW, Chou MY, Iijima K, et al. Asian Working Group for Sarcopenia: 2019 Consensus Update on Sarcopenia Diagnosis and Treatment. J Am Med Dir Assoc. 2020;21:300-7 e2.

17. Rubenstein LZ, Harker JO, Salva A, Guigoz Y, Vellas B. Screening for undernutrition in geriatric practice: developing the short-form mini-nutritional assessment (MNA-SF). J Gerontol A Biol Sci Med Sci. 2001;56:M366-72.

18. Folstein MF, Folstein SE, McHugh PR. "Mini-mental state". A practical method for grading the cognitive state of patients for the clinician. J Psychiatr Res. 1975;12:189-98.

19. Holland AE, Spruit MA, Troosters T, Puhan MA, Pepin V, Saey D, et al. An official European Respiratory Society/American Thoracic Society technical standard: field walking tests in chronic respiratory disease. Eur Respir J. 2014;44:1428-46.

20. Ward RE, Leveille SG, Beauchamp MK, Travison T, Alexander N, Jette AM, et al. Functional performance as a predictor of injurious falls in older adults. J Am Geriatr Soc. 2015;63:315-20.

21. Tey S, Huynh D, Berde Y, Baggs G, How C, Low Y, et al. Prevalence of low muscle mass and associated factors in community-dwelling older adults in Singapore. Scientific reports. 2021;11:23071.
