# Supplementary material for: Does high-frequency resistance exercise offer additional benefits to older adults? learnings from a randomized controlled trial
Source: BMC Sports Sci Med Rehabil. 2024 Sep 6;16:186. doi: 10.1186/s13102-024-00975-6 (PMC11378542; doi:10.1186/s13102-024-00975-6)
Supplement: Supplementary file 3 — Supplementary Material 3 [file 13102_2024_975_MOESM3_ESM.docx]

**Supplementary Tables**

Supplementary Table 1. Sensitivity analysis of older adults completed the study and who withdrew from the study

| Variable | Within (N=127) | Withdrew (N=22) | *p* |
| --- | --- | --- | --- |
| Age (yr) | 71.96±4.54 | 73.64±4.10 | 0.108 |
| Height (cm) | 162.90±8.50 | 160.68±7.59 | 0.253 |
| Weight (kg) | 62.92±10.11 | 62.70±9.45 | 0.926 |
| Waist hip ratio | 0.92±0.07 | 0.94±0.07 | 0.081 |
| Systolic blood pressure (mmHg) | 132.57±19.55 | 132.50±16.52 | 0.987 |
| Diastolic blood pressure (mmHg) | 75.98±11.43 | 73.68±8.82 | 0.372 |
| MNA-SF sore | 12.94±1.38 | 13.41±0.91 | 0.126 |
| Lymphocyte count (10^9^/L) | 2.18±0.87 | 2.07±0.46 | 0.583 |
| Prealbumin (g/L) | 266.02±37.67 | 273.02±42.01 | 0.468 |
| Gender (male) | 62(48.80) | 8(36.4) | 0.357 |
| Living alone | 23(18.1) | 4(18.2) | 1.000 |
| House with stairs | 61(48.0) | 13(59.1) | 0.365 |
| Smoking | 20(15.7) | 3(13.6) | 1.000 |
| Alcohol consumption | 8(6.3) | 2(9.1) | 0.335 |
| Hypertension | 72(56.7) | 14(63.6) | 0.643 |
| Diabetes mellitus | 40(31.5) | 6(27.3) | 0.806 |
| Falls history | 14(11.0) | 4(18.2) | 0.491 |
| Medication |  |  |  |
| Statin | 46(36.2) | 12(54.5) | 0.154 |
| Metformin | 17(13.4) | 6(27.3) | 0.213 |
| Insulin | 7(5.5) | 0(0.0) | 0.597 |

MNA-SF, mini nutritional assessment short-form.

Supplementary Table 2. Absolute changes in muscle parameters and body composition after dose-different exercise

|  | Control (male 19, female 21) | | | |  | Low-dose (male 24, female 22) | | | |  | High-dose (male 19, female 22) | | | |
| --- | --- | --- | --- | --- | --- | --- | --- | --- | --- | --- | --- | --- | --- | --- |
| Characteristics | Baseline | Follow-up | Changes | *p* |  | Baseline | Follow-up | Changes | *p* |  | Baseline | Follow-up | Changes | *p* |
| grip strength (kg) | 27.17±9.74 | 26.47±9.91 | -0.69±3.35 | 0.199 |  | 27.98±9.71 | 28.87±9.90 | 0.89±3.18 | 0.065 |  | 26.53±9.33 | 28.69±9.84 | 2.17±3.64 | <0.001 |
| 5 times sit to stand test (s) | 7.83±1.45 | 8.68±2.91 | 0.84±2.78 | 0.062 |  | 8.10±1.42 | 7.56±1.40 | -0.53±1.37 | 0.011 |  | 8.68±1.87 | 7.02±1.19 | -1.66±1.42 | <0.001 |
| gait speed (m/s) | 1.43±0.22 | 1.48±0.26 | 0.04±0.25 | 0.276 |  | 1.47±0.25 | 1.58±0.24 | 0.11±0.27 | 0.008 |  | 1.38±0.25 | 1.54±0.29 | 0.16±0.31 | 0.002 |
| SPPB score | 11.50±0.72 | 11.65±0.62 | 0.15±0.80 | 0.244 |  | 11.61±0.86 | 11.93±0.25 | 0.33±0.79 | 0.008 |  | 11.34±0.91 | 11.85±0.36 | 0.51±0.93 | 0.001 |
| 6MWT (m) | 468.60±58.45 | 469.83±55.38 | 1.23±49.89 | 0.877 |  | 477.41±59.88 | 503.13±55.26 | 25.72±42.89 | <0.001 |  | 464.66±63.49 | 503.44±50.10 | 38.78±49.58 | <0.001 |
| Skeletal muscle mass (kg) | 24.15±5.54 | 23.78±5.58 | -0.36±0.67 | 0.001 |  | 24.05±4.63 | 24.23±4.63 | 0.17±0.74 | 0.118 |  | 23.16±4.53 | 23.82±4.58 | 0.66±0.76 | <0.001 |
| ASMI (kg/m^2^) | 6.94±1.12 | 6.75±1.08 | -0.19±0.21 | <0.001 |  | 6.86±0.94 | 6.80±0.89 | -0.06±0.23 | 0.098 |  | 6.61±0.92 | 6.66±0.92 | 0.05±0.21 | 0.106 |
| Phase angle (°) | 4.69±0.56 | 4.79±0.58 | 0.10±0.25 | 0.017 |  | 4.76±0.53 | 4.78±0.55 | 0.03±0.33 | 0.566 |  | 4.64±0.61 | 4.71±0.57 | 0.07±0.30 | 0.164 |
| Fat mass (kg) | 19.24±4.79 | 19.31±4.72 | 0.07±1.44 | 0.761 |  | 18.37±5.87 | 17.11±5.67 | -1.26±1.87 | <0.001 |  | 19.38±5.85 | 17.73±5.39 | -1.65±1.87 | <0.001 |
| Fat mass index (kg/m2) | 7.37±1.85 | 7.41±1.85 | 0.04±0.56 | 0.635 |  | 7.01±2.63 | 6.52±2.47 | -0.5±0.72 | <0.001 |  | 7.31±2.36 | 6.68±2.13 | -0.63±0.76 | <0.001 |
| Visceral fat area (cm^2^) | 91.07±27.68 | 92.44±28.34 | 1.37±8.49 | 0.313 |  | 88.65±34.61 | 81.12±32.46 | -7.54±11.16 | <0.001 |  | 94.24±31.83 | 84.22±27.77 | -10.01±12.72 | <0.001 |

SPPB, short physical performance battery; 6MWT, 6-minute walking test; ASMI, appendicular skeletal muscle mass index.

Supplementary Table 3. Between-group differences in changes of muscle parameters and body composition

|  | Low-dose versus Control | | | | High-dose versus Control | | | | High-dose versus Low-dose | | | |
| --- | --- | --- | --- | --- | --- | --- | --- | --- | --- | --- | --- | --- |
| Changes from baseline to 24-week | Mean difference (95% CI) | *p* | Adjusted mean difference (95% CI) | *p* | Mean difference (95% CI) | *p* | Adjusted mean difference (95% CI) | *p* | Mean difference (95% CI) | *p* | Adjusted mean difference (95% CI) | *p* |
| grip strength (kg) | 1.58(0.13,3.03) | 0.033 | 1.61(0.16,3.06) | 0.030 | 2.86(1.36,4.35) | <0.001 | 2.84(1.35,4.33) | <0.001 | 1.28(-0.16,2.72) | 0.081 | 1.23(-0.21,2.68) | 0.093 |
| 5 times sit to stand test (s) | -1.38(-2.21,-0.55) | 0.001 | -1.25(-2.02,-0.48) | 0.002 | -2.50(-3.32,-1.63) | <0.001 | -2.09(-2.89,-1.29) | <0.001 | -1.12(-1.95,-0.30) | 0.008 | -0.84(-1.61,-0.07) | 0.033 |
| gait speed (m/s) | 0.07(-0.05,0.19) | 0.274 | 0.09(-0.02,0.19) | 0.096 | 0.12(-0.003,0.24) | 0.060 | 0.09(-0.02,0.20) | 0.120 | 0.05(-0.07,0.17) | 0.395 | -0.003(-0.11,0.10) | 0.952 |
| SPPB score | 0.18(-0.18,0.54) | 0.334 | 0.27(0.09,0.45) | 0.003 | 0.36(0.002,0.73) | 0.055 | 0.22(0.04,0.41) | 0.018 | 0.19(-0.17,0.54) | 0.304 | -0.05(-0.23,0.13) | 0.603 |
| 6MWT (m) | 24.50(4.23,44.76) | 0.018 | 28.13(10.83,45.44) | 0.002 | 37.56(16.72,58.39) | <0.001 | 35.93(18.16,53.69) | <0.001 | 13.06(-7.07,33.20) | 0.202 | 7.80(-9.43,25.02) | 0.372 |
| Skeletal muscle mass (kg) | 0.54(0.22,0.83) | 0.001 | 0.54(0.23,0.85) | 0.001 | 1.02(0.71,1.34) | <0.001 | 1.02(0.70,1.34) | <0.001 | 0.49(0.18,0.79) | 0.002 | 0.48(0.17,0.79) | 0.003 |
| ASMI (kg/m2) | 0.13(0.03,0.22) | 0.006 | 0.13(0.04,0.22) | 0.006 | 0.24(0.14,0.33) | <0.001 | 0.22(0.13,0.32) | <0.001 | 0.11(0.02,0.20) | 0.020 | 0.10(0.01,0.19) | 0.036 |
| Phase angle (°) | -0.07(-0.20,0.06) | 0.267 | -0.06(-0.19,0.06) | 0.316 | -0.03(-0.16,0.10) | 0.607 | -0.04(-0.17,0.09) | 0.522 | 0.04(-0.09,0.16) | 0.558 | 0.02(-0.10,0.14) | 0.729 |
| Fat mass (kg) | -1.33(-2.08,-0.58) | 0.001 | -1.41(-2.13,-0.69) | <0.001 | -1.72(-2.48,-0.95) | <0.001 | -1.70(-2.44,-0.97) | <0.001 | -0.39(-1.13,0.35) | 0.303 | -0.29(-1.01,0.42) | 0.417 |
| Fat mass index (kg/m2) | -0.54(-0.84,-0.25) | <0.001 | -0.58(-0.86,-0.30) | <0.001 | -0.68(-0.98,-0.38) | <0.001 | -0.68(-0.97,-0.40) | <0.001 | -0.13(-0.43,0.16) | 0.374 | -0.10(-0.38,0.17) | 0.465 |
| Visceral fat area (cm2) | -8.91(-13.60,-4.22) | <0.001 | -9.20(-13.62,-4.78) | <0.001 | -11.39(-16.20,-6.57) | <0.001 | -11.01(-15.56,-6.46) | <0.001 | -2.47(-7.13,2.18) | 0.295 | -1.81(-6.21,2.60) | 0.418 |

SPPB, short physical performance battery; 6MWT, 6-minute walking test; ASMI, appendicular skeletal muscle mass index; CI, confidence interval. Adjusted mean difference, adjusted by baseline levels.

Supplementary Table 4. Between-group differences of female participants in changes of muscle parameters and body composition

|  | Low-dose versus Control | | | | High-dose versus Control | | | | High-dose versus Low-dose | | | |
| --- | --- | --- | --- | --- | --- | --- | --- | --- | --- | --- | --- | --- |
| Change from baseline to 24-week | Mean difference (95% CI) | *p* | Adjusted mean difference (95% CI) | *p* | Mean difference (95% CI) | *p* | Adjusted mean difference (95% CI) | *p* | Mean difference (95% CI) | *p* | Adjusted mean difference (95% CI) | *p* |
| grip strength (kg) | 1.46(-0.28,3.19) | 0.098 | 1.58(-0.12,3.28) | 0.067 | 2.63(0.89,4.37) | 0.004 | 2.79(1.09,4.49) | 0.002 | 1.17(-0.54,2.89) | 0.175 | 1.21(-0.46,2.88) | 0.155 |
| 5 times sit to stand test (s) | -1.07(-2.33,0.19) | 0.095 | -1.02(-2.26,0.23) | 0.107 | -2.37(-3.63,-1.1) | <0.001 | -2.03(-3.33,-0.72) | 0.003 | -1.30(-2.55,-0.05) | 0.041 | -1.01(-2.28,0.26) | 0.118 |
| gait speed (m/s) | 0.15(0.01,0.28) | 0.038 | 0.15(0.04,0.26) | 0.007 | 0.19(0.04,0.32) | 0.009 | 0.15(0.04,0.26) | 0.010 | 0.04(-0.10,0.18) | 0.560 | -0.01(-0.12,0.11) | 0.924 |
| SPPB score | 0.08(-0.47,0.63) | 0.779 | 0.35(0.04,0.66) | 0.030 | 0.31(-0.25,0.86) | 0.273 | 0.30(-0.01,0.61) | 0.061 | 0.23(-0.32,0.77) | 0.408 | -0.05(-0.36,0.26) | 0.733 |
| 6MWT (m) | 23.68(-2.02,49.37) | 0.070 | 31.56(9.34,53.77) | 0.006 | 48.72(23.03,74.42) | <0.001 | 47.97(25.98,69.95) | <0.001 | 25.05(-0.35,50.44) | 0.053 | 16.41(-5.60,38.42) | 0.141 |
| Skeletal muscle mass (kg) | 0.62(0.22,1.02) | 0.003 | 0.62(0.22,1.02) | 0.003 | 1.19(0.79,1.59) | <0.001 | 1.19(0.80,1.60) | <0.001 | 0.58(0.18,0.97) | 0.005 | 0.58(0.18,0.97) | 0.005 |
| ASMI(kg/m2) | 0.17(0.03,0.30) | 0.016 | 0.17(0.03,0.30) | 0.015 | 0.27(0.13,0.40) | <0.001 | 0.26(0.13,0.40) | <0.001 | 0.10(-0.03,0.23) | 0.139 | 0.10(0.04,0.23) | 0.148 |
| Phase angle (°) | -0.12(-0.29,0.07) | 0.193 | -0.11(-0.28,0.07) | 0.234 | 0.11(-0.07,0.29) | 0.217 | 0.10(-0.08,0.28) | 0.270 | 0.23(0.05,0.40) | 0.012 | 0.21(0.03,0.39) | 0.025 |
| Fat mass (kg) | -1.86(-2.96,-0.76) | 0.001 | -1.70(-2.79,-0.62) | 0.003 | -2.12(-3.22,-1.01) | <0.001 | -2.04(-3.12,-0.97) | <0.001 | -0.25(-1.34,0.83) | 0.642 | -0.34(-1.41,0.72) | 0.523 |
| Fat mass index (kg/m2) | -0.80(-1.26,-0.34) | 0.001 | -0.74(-1.18,-0.30) | 0.001 | -0.88(-1.35,-0.43) | <0.001 | -0.88(-1.32,-0.44) | <0.001 | -0.09(-0.54,0.36) | 0.689 | -0.14(-0.58,0.30) | 0.520 |
| Visceral fat area (cm2) | -13.52(-21.14,-5.90) | 0.001 | -12.35(-19.87,-4.84) | 0.002 | -15.95(-23.57,-8.33) | <0.001 | -15.28(-22.74,-7.83) | <0.001 | -2.43(-9.96,5.10) | 0.521 | -2.93(-10.29,4.43) | 0.429 |

SPPB, short physical performance battery; 6MWT, 6-minute walking test; ASMI, appendicular skeletal muscle mass index; CI, confidence interval. Adjusted mean difference, adjusted by baseline levels.

Supplementary Table 5. Between-group differences of male participants in changes of muscle parameters and body composition

|  | Low-dose versus Control | | | | High-dose versus Control | | | | High-dose versus Low-dose | | | |
| --- | --- | --- | --- | --- | --- | --- | --- | --- | --- | --- | --- | --- |
| Change from baseline to 24-week | Mean difference (95% CI) | *p* | Adjusted mean difference (95% CI) | *p* | Mean difference (95% CI) | *p* | Adjusted mean difference (95% CI) | *p* | Mean difference (95% CI) | *p* | Adjusted mean difference (95% CI) | *p* |
| grip strength (kg) | 1.71(-0.71,4.14) | 0.162 | 1.68(-0.76,4.11) | 0.172 | 3.11(0.54,5.67) | 0.018 | 3.00(0.41,5.59) | 0.024 | 1.39(-1.03,3.82) | 0.255 | 1.32(-1.13,3.76) | 0.285 |
| 5 times sit to stand test (s) | -1.74(-2.84,-0.64) | 0.002 | -1.49(-2.42,-0.57) | 0.002 | -2.62(-3.79,-1.46) | <0.001 | -2.31(-3.29,-1.34) | <0.001 | -0.89(-1.98,0.21) | 0.112 | -0.82(-1.74,0.10) | 0.078 |
| gait speed (m/s) | -0.02(-0.22,0.18) | 0.852 | 0.02(-0.16,0.19) | 0.869 | 0.04(-0.1,0.25) | 0.706 | 0.01(-0.18,0.20) | 0.911 | 0.05(-0.14,0.26) | 0.559 | -0.004(-0.19,0.18) | 0.964 |
| SPPB score | 0.29(-0.19,0.77) | 0.226 | 0.18(0.01,0.37) | 0.061 | 0.42(-0.08,0.93) | 0.100 | 0.14(0.07,0.34) | 0.181 | 0.12(-0.35,0.61) | 0.590 | -0.04(-0.24,0.15) | 0.646 |
| 6MWT (m) | 25.03(-7.28,57.33) | 0.126 | 23.88(-3.28,51.05) | 0.084 | 24.68(-9.48,58.82) | 0.153 | 22.32(-6.39,51.03) | 0.125 | -0.34(-32.65,31.96) | 0.983 | -1.56(-28.73,25.60) | 0.909 |
| Skeletal muscle mass (kg) | 0.43(-0.06,0.93) | 0.081 | 0.36(-0.13,0.84) | 0.147 | 0.83(0.31,1.35) | 0.002 | 0.71(0.19,1.23) | 0.009 | 0.39(-0.10,0.89) | 0.113 | 0.35(-0.13,0.83) | 0.150 |
| ASMI (kg/m2) | 0.09(-0.04,0.23) | 0.148 | 0.06(-0.06,0.19) | 0.320 | 0.21(0.07,0.35) | 0.004 | 0.14(0.002,0.28) | 0.046 | 0.11(-0.02,0.24) | 0.090 | 0.08(-0.05,0.20) | 0.221 |
| Phase angle (°) | -0.04(-0.21,0.14) | 0.677 | -0.04(-0.20,0.12) | 0.626 | -0.20(-0.39,-0.01) | 0.036 | -0.19(-0.36,-0.03) | 0.025 | -0.16(-0.34,0.01) | 0.069 | -0.16(-0.32,0.01) | 0.057 |
| Fat mass (kg) | -0.79(-1.83,0.25) | 0.135 | -1.23(-2.27,-0.19) | 0.022 | -1.27(-2.37,-0.17) | 0.024 | -1.37(-2.41,-0.32) | 0.012 | -0.48(-1.52,0.56) | 0.357 | -0.14(-1.16,0.89) | 0.791 |
| Fat mass index (kg/m2) | -0.29(-0.67,0.09) | 0.129 | -0.48(-0.84,-0.12) | 0.009 | -0.43(-0.84,-0.04) | 0.032 | -0.49(-0.85,-0.12) | 0.009 | -0.15(-0.52,0.23) | 0.438 | -0.01(-0.36,0.34) | 0.969 |
| Visceral fat area (cm2) | -4.33(-9.63,0.98) | 0.108 | -6.94(-11.95,-1.92) | 0.008 | -6.19(-11.80,-0.59) | 0.031 | -6.50(-11.58,-1.41) | 0.013 | -1.87(-7.17,3.44) | 0.484 | 0.43(-4.53,5.40) | 0.861 |

SPPB, short physical performance battery; 6MWT, 6-minute walking test; ASMI, appendicular skeletal muscle mass index; CI, confidence interval. Adjusted mean difference, adjusted by baseline levels.
